# Mice lacking C1q or C3 show accelerated rejection of minor H disparate skin grafts and resistance to induction of tolerance

**DOI:** 10.1002/eji.200940158

**Published:** 2010-03-08

**Authors:** Paramita Baruah, Elizabeth Simpson, Ingrid E Dumitriu, Katy Derbyshire, David Coe, Caroline Addey, Julian Dyson, Jian-Guo Chai, Terence Cook, Diane Scott, Marina Botto

**Affiliations:** 1 Rheumatology Section, Faculty of Medicine, Imperial College LondonHammersmith Campus, London, UK; 2 Department of Immunology, Faculty of Medicine, Imperial College LondonHammersmith Campus, London, UK; 3 Department of Histopathology, Faculty of Medicine, Imperial College LondonHammersmith Campus, London, UK

**Keywords:** Complement, Skin graft, Tolerance

## Abstract

Complement activation is known to have deleterious effects on organ transplantation. On the other hand, the complement system is also known to have an important role in regulating immune responses. The balance between these two opposing effects is critical in the context of transplantation. Here, we report that female mice deficient in C1q (C1qa^−/−^) or C3 (C3^−/−^) reject male syngeneic grafts (HY incompatible) at an accelerated rate compared with WT mice. Intranasal HY peptide administration, which induces tolerance to syngeneic male grafts in WT mice, fails to induce tolerance in C1qa^−/−^ or C3^−/−^ mice. The rejection of the male grafts correlated with the presence of HY D^b^*Uty*-specific CD8^+^ T cells. Consistent with this, peptide-treated C1qa^−/−^ and C3^−/−^ female mice rejecting male grafts exhibited more antigen-specific CD8^+^IFN-γ^+^ and CD8^+^IL-10^+^ cells compared with WT females. This suggests that accumulation of IFN-γ- and IL-10-producing T cells may play a key role in mediating the ongoing inflammatory process and graft rejection. Interestingly, within the tolerized male skin grafts of peptide-treated WT mice, IFN-γ, C1q and C3 mRNA levels were higher compared to control female grafts. These results suggest that C1q and C3 facilitate the induction of intranasal tolerance.

## Introduction

Most of the research undertaken so far to decipher the role of the immune response in allograft rejection and tolerance has focused predominantly on adaptive T-cell-mediated responses. Recently, the role of the innate immune system in this field has been more closely examined, following the discovery that the innate system has a critical role in shaping the adaptive immune responses. The complement system is an integral component of innate immunity with well-established roles in modulation of humoral immune responses. It has, however, become increasingly evident in recent years that the complement system regulates antigen-specific T-cell responses as well 1,2. Complement can both enhance T-cell immune responses to infectious agents and mediate immunosuppression and antigen tolerance 3,4. As cytotoxic effector T cells are instrumental in mediating graft rejection, it follows that complement may impact on graft rejection *via* its effect on T cells. Traditionally, complement activation is associated with tissue injury and inflammation that occur during graft rejection. Deposition of complement activation components such as C3, C3d and C4d is noted in transplanted organs that have been rejected 5,6, but MHC-mismatched transplants also induce antibody responses that could account for these findings.

Another common phenomenon in transplanted organs is ischemia/reperfusion injury that induces complement activation, which could contribute to the pathogenic mechanisms that culminate in graft rejection 7,8. Pratt *et al*. have elegantly demonstrated that intrarenal production of C3 is essential for graft rejection in a renal allograft model 9. The above studies have mainly explored the impact of complement on MHC-incompatible allografts. Less is known about the role of complement, especially the components C1q and C3, in the T-cell-only response to minor histocompatibility disparate grafts such as HY, the male-specific minor antigens.

C1q is the first component of the classical complement pathway and is best known for its binding to immune complexes and activation of the classical pathway 10,11. The classical complement pathway converges with the alternative and lectin pathways at the level of C3, from where effector functions of complement are generated. Both C1q and C3 have been shown to impact on the function of antigen presenting cells and T cells *in vitro* and *in vivo* 12–15. Interestingly, impaired T-cell responses are reported in mice lacking complement in several disease models including autoimmune disease and transplantation 16–18.

Syngeneic male skin grafts are rejected by female mice of high responder H2^b^ strains such as C57BL/6 (B6) and this is associated with the development of H2-restricted cell responses by CD4^+^ and CD8^+^ T cells, with rejection mediated predominantly by cytotoxic CD8^+^ effectors 19–23. Prolonged survival of syngeneic male skin grafts can be induced by intranasal administration of HY peptides to B6 female mice 24. Here, we report a hitherto unknown effect of C1q and C3 on both the survival of HY-disparate skin grafts and on the induction of transplantation tolerance to such grafts by intranasal administration of HY peptides. Our data show that both C1q and C3 in recipients have protective functions in the survival of male skin grafted on female mice and are required for the induction of tolerance following intranasal peptide administration. The findings also suggest that the lack of C1q or C3 prevents induction of intranasal tolerance by modulating local IFN-γ production.

## Results

### C1q-deficient and C3-deficient female mice reject male skin grafts faster than WT mice

**Figure 1:**
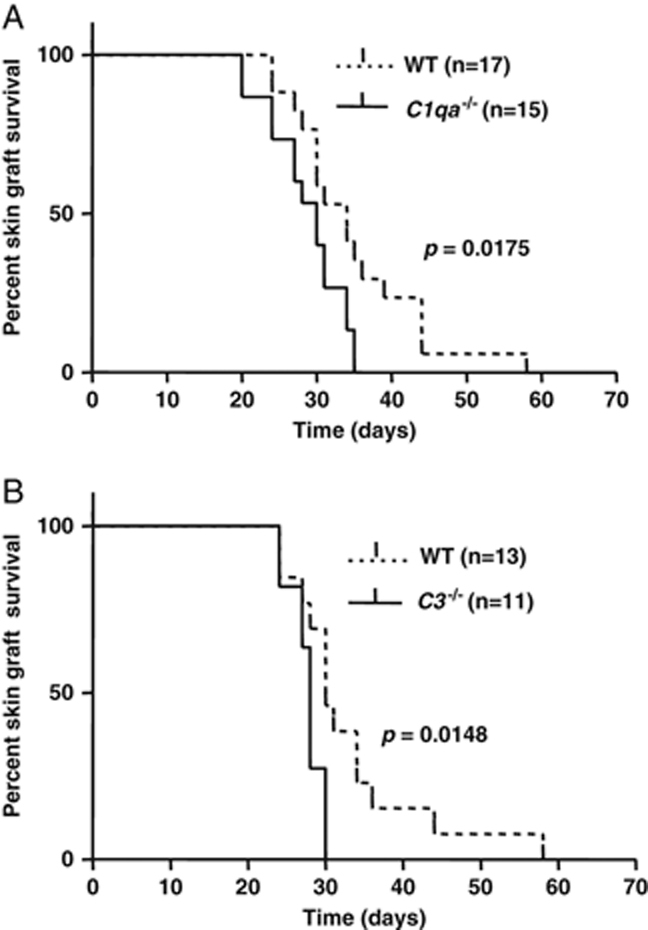
Accelerated rejection of syngeneic male grafts by C1qa^−/−^ and C3^−/−^ female mice. WT, C1qa^−/−^ and C3^−/−^ female mice were grafted with skin from WT, C1qa^−/−^ and C3^−/−^ male mice, respectively, and graft rejection monitored. (A) Survival of syngeneic male skin grafts transplanted on female WT (*n*=17) and C1qa^−/−^ (*n*=15) mice. Data shown are pooled results from three independent experiments (*p*=0.0175). (B) Survival of syngeneic male grafts transplanted on female WT (*n*=13) and C3^−/−^ (*n*=11) mice. Data shown are pooled results from two independent experiments (*p*=0.0148). Statistical analysis was performed using the log-rank method for survival curves.

To investigate the role of C1q and C3 in graft rejection, we used a syngeneic model in which female mice were grafted with tail skin from syngeneic male mice and the rejection of the graft was monitored. As a control, we grafted tail skin taken from female mice in the same graft bed as the male. When WT mice were used as donors and recipients, while all the female recipient mice retained the grafted female tissue indefinitely, the male grafts were rejected with a mean survival time (MST) of 35 days. We first evaluated whether the lack of C1q or C3 influenced the rejection of male skin grafts. WT, C1qa^−/−^ and C3^−/−^ female mice were grafted with WT, C1qa^−/−^ and C3^−/−^ male skin, respectively. Control WT, C1qa^−/−^ and C3^−/−^ female skin grafts were also placed in the same graft bed. Interestingly, C1qa^−/−^ and C3^−/−^ female mice rejected the male skin grafts significantly faster than the WT female mice (Fig. 1A and B). The control female skin grafts were retained indefinitely in all three groups of mice (data not shown), indicating genetic homogeneity within each strain, with appropriate number of backcross generations of the mutant C1qa^−/−^ and C3^−/−^ gene-targeted strains to B6.

### Rejection of allogenic skin grafts

We next evaluated whether male skin lacking C1q or C3 would elicit a different pattern of rejection on WT female recipients than WT male skin. WT, C1qa^−/−^ or C3^−/−^ test male and control female tail skin grafts were given to groups of female WT mice. WT, C1qa^−/−^ and C3^−/−^ female tail skin was co-grafted as control. Lack of C1q or C3 in the donor skin did not significantly influence the rate of rejection of the male grafts (data not shown).

We then explored whether the rejection pattern of MHC-mismatched allogenic grafts was influenced by the absence of C1q or C3. For this purpose, B6 WT, B6.C1qa^−/−^ and B6.C3^−/−^ female mice were, respectively, given BALB/c WT, BALB/c.C1qa^−/−^ and BALB/c.C3^−/−^ female skin grafts. As expected, the rejection of the MHC-disparate allogenic skin graft followed a faster time course (8–10 days) than that of syngeneic male skin grafts. However, no differences were observed among the groups (data not shown). In addition, we transplanted female B6 mice with BALB/c WT, BALB/c.C1qa^−/−^ or BALB/c.C3^−/−^ female skin grafts, and again the rejection rates of MHC-disparate allogenic grafts were similar (data not shown).

### C1q- and C3-deficient female mice fail to show tolerance induction by intranasal HY peptide

**Figure 2:**
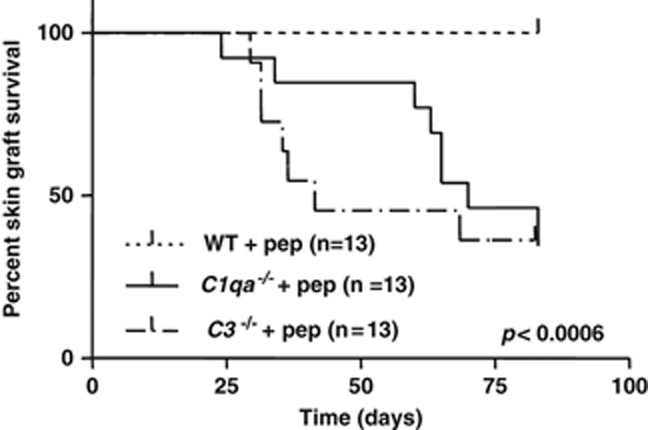
Failure of induction of peptide tolerance in C1qa^−/−^ and C3^−/−^ female mice. WT, C1qa^−/−^ and C3^−/−^ female mice were given HY^Db^*Uty* peptide (100 μg for 3 days) intranasally to induce tolerance. Survival of syngeneic male skin grafts transplanted on peptide-treated female WT (*n*=13), C1qa^−/−^ (*n*=13) and C3^−/−^ (*n*=13) mice (pooled results from two independent experiments) (WT *versus* C1qa^−/−^ and WT *versus* C3^−/−^ mice: *p*<0.0006). Statistical analysis was performed using the log-rank method.

Tolerance to male skin grafts can be induced by intranasal administration of H2-A^b^ restricted *Dby*-encoded HY peptide to WT mice. As previously reported 24, we administered 100 μg of peptide intranasally to WT mice daily for 3 days. Peptide pretreated and control untreated mice were grafted 10 days later with syngeneic male and female WT skin. WT female mice that received the HY peptide intranasally prior to grafting, retained male skin grafts indefinitely (Fig. 2) while control untreated female mice rejected the male skin with a MST of 35 days (data not shown). In both groups of WT mice, the syngeneic female skin grafts were retained indefinitely. In contrast, both C1qa^−/−^ and C3^−/−^ female mice given the intranasal peptide failed to develop tolerance to the male skin, with the grafts being rejected with an MST of 70 and 65 days respectively (Fig. 2).

### Antigen-specific tetramer-positive Uty^+^CD8^+^ T cells in circulation

**Figure 3:**
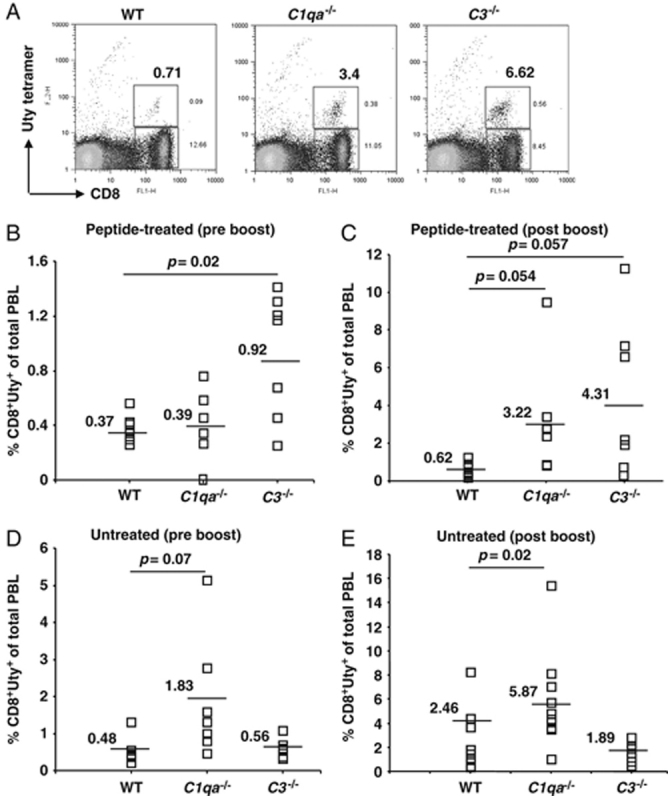
HY antigen-specific Uty^+^CD8^+^ T cells in peripheral blood. Female WT, C1qa^−/−^ and C3^−/−^ mice given intranasal peptide 10 days before grafting with syngeneic male skin 10 wk previously were tail-bled before and 7 days after boosting with intraperitoneal male splenocytes. At this time, WT mice were tolerant of their male grafts, while mice in both complement-deficient groups had rejected theirs. PBL were stained with mAb against CD8 and with Uty tetramers. (A) FACS profiles of PBL from one mouse in each group stained *ex vivo* with anti-CD8 and Uty tetramers 7 days after the intraperitoneal boost. (B, C) Percentage of CD8^+^Uty^+^ T cells in PBL of each mouse in the three groups before (B), and after (C), intraperitoneal boosting with male splenocytes. *n*=6–7 each group. (D, E) Percentage of CD8^+^Uty^+^ T cells in PBL of non-peptide-treated WT, C1qa^−/−^ and C3^−/−^ mice subsequently grafted with syngeneic male skin before (*n*=7 each group) and after intraperitoneal boosting (*n*=9, 11, 7 respectively; pooled results of two experiments) with male splenocytes. Each symbol represents data from an individual mouse, horizontal bars and the numbers next to the bars indicate the mean (Statistical analysis with Student's *t*-test).

The frequencies of antigen-specific CD8^+^ T cells were evaluated *ex vivo* 10 wk after grafting with syngeneic male skin in the WT, C1qa^−/−^ and C3^−/−^ females pretreated with intranasal peptide. At this time point, the WT mice retained their test male skin graft, whereas the complement-deficient mice had rejected theirs. Figure 3A shows the percentage of Uty-tetramer-positive CD8^+^ T after HY boost by intraperitoneal injection of male spleen cells. Before boosting, low frequencies of Uty-tetramer-positive CD8^+^ T cells were identified in the peripheral blood of the peptide-treated WT and C1qa^−/−^ mice, while C3^−/−^ recipients had slightly increased frequencies of circulating Uty-tetramer-positive CD8^+^ T cells (Fig. 3B). Following a booster intraperitoneal injection of 5×10^6^ male spleen cells, the level of Uty-tetramer-positive CD8^+^ T cells in the peripheral blood increased in both groups of complement-deficient females, but not in the WT group (Fig. 3A and C). Control non-peptide treated groups of females grafted with WT, C1qa^−/−^ and C3^−/−^ male skin were similarly tested before (Fig. 3D), and after boosting, 8–10 wk after grafting (Fig. 3E). After injection of male spleen cells, the levels of Uty-tetramer-positive CD8^+^ T cells in each group were increased, most markedly in the C1q^−/−^ mice (Fig. 3E).

### Ex vivo cytokine responses following intranasal peptide treatment

**Figure 4:**
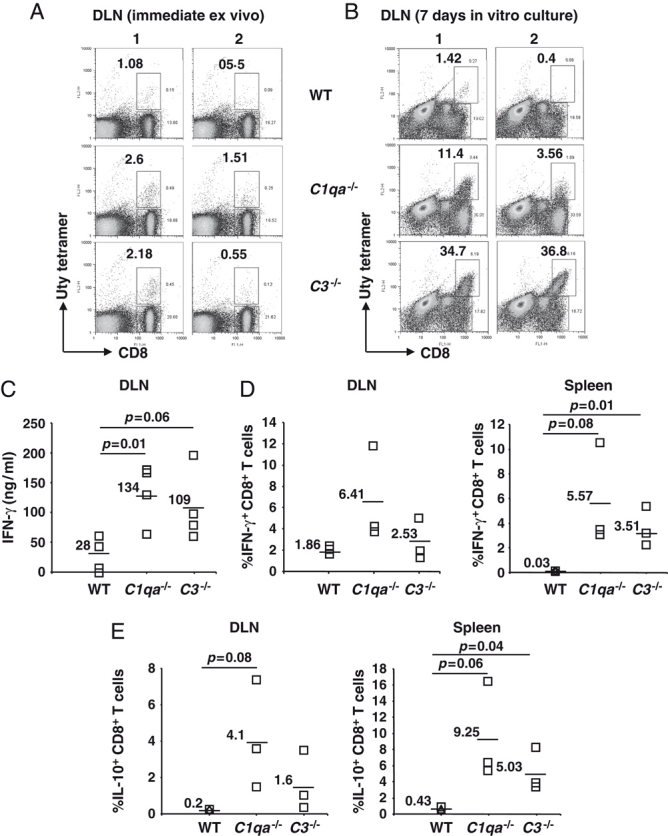
*Ex vivo* cytokine responses following intranasal peptide and skin grafting. HY^Ab^*Dby* peptide was administered intranasally to WT, C1qa^−/−^ and C3^−/−^ mice followed by grafting of WT, C1qa^−/−^ and C3^−/−^ male skin respectively ten days later. Following an intraperitoneal boost with male splenocytes 12 wk later, the mice were sacrificed and cells from DLN and spleen harvested. The cells were re-stimulated *in vitro* with irradiated male splenocytes and stained with anti-CD8 mAb and Uty-tetramer. (A, B) Representative FACS profiles of Uty-tetramer and CD8 on cells of the DLN before and after *in vitro* restimulation. (C) Cells isolated from DLN were co-cultured with irradiated female or male splenocytes and IFN-γ levels were quantified by ELISA. Each symbol represents data from an individual mouse as the difference in IFN-γ produced following *in vitro* stimulation with male splenocytes *versus* female splenocytes. (D) Cells isolated from the DLN and spleen were cultured *in vitro* with irradiated male splenocytes and restimulated with HY^Db^*Uty-*peptide-pulsed female splenocytes. IFN-γ-producing CD8^+^ T cells were quantified by intracellular staining and flow cytometry. (E) Cells from DLN and spleen were cultured *in vitro* with irradiated male splenocytes and then restimulated with HY^Db^*Uty-*peptide-pulsed female splenocytes. IL-10-producing CD8^+^ T cells were quantified by intracellular staining and flow cytometry. (C–E) Results are pooled from two independent experiments; horizontal bars and the numbers next to the bars indicate the mean (statistical analysis with Student's *t*-test).

Female mice (WT, C1qa^−/−^ and C3^−/−^) pre-treated with intranasal peptide before being grafted with syngeneic male skin (from WT, C1qa^−/−^ and C3^−/−^ donors, respectively) were boosted (intraperitoneally) with syngeneic male splenocytes 12 wk after grafting, at a time when mice in the WT group were tolerant of their skin grafts, whereas the complement-deficient mice had rejected theirs (see Fig. 2). Five days later, the mice were sacrificed and DLN were analyzed for the presence of antigen-specific CD8^+^ T cells. A higher percentage of tetramer-positive Uty^+^CD8^+^ T cells was found in the DLN of C1qa^−/−^ and C3^−/−^ mice than in WT (Fig. 4A). Moreover, following 7 days of *in vitro* culture with irradiated male splenocytes, the tetramer-positive Uty^+^CD8^+^ T cells from DLN of the C1qa^−/−^ and C3^−/−^ mice exhibited a significant expansion (up to 67-fold) (Fig. 4B). In contrast, the Uty^+^CD8^+^ T cells from DLN of WT mice failed to expand (Fig. 4B).

The supernatants from these co-cultures of cells from the DLN of C1qa^−/−^ and C3^−/−^ mice were found to have significantly higher levels of IFN-γ than those from WT mice (Fig. 4C). Furthermore, intracellular staining confirmed that the CD8^+^ T cells from the DLN and spleen cultures produced IFN-γ in an antigen-dependent manner (Fig. 4D). Interestingly, C1qa^−/−^ and C3^−/−^ mice also displayed significantly higher percentages of antigen induced CD8^+^IL-10^+^ T cells than WT mice (Fig. 4E). Very low percentages of the CD4^+^ T cells from the DLN or spleen produced IL-10, but the trend observed in the three groups of mice was similar to that of the CD8^+^ T cells (data not shown). In the groups of mice that had not received intranasal peptide prior to grafting, all of which had rejected the test male skin grafts, the percentages of CD8^+^IL-10^+^ T cells were found to be increased both in the DLN and spleen of the C1qa^−/−^ and C3^−/−^ mice in comparison with WT mice. In contrast, the percentages of CD8^+^IFN-γ^+^ T cells did not differ significantly between the groups (Supporting Information Fig. 1).

### Tolerized male skin grafts express increased levels of C1q, C3 and IFN-γ

**Figure 5:**
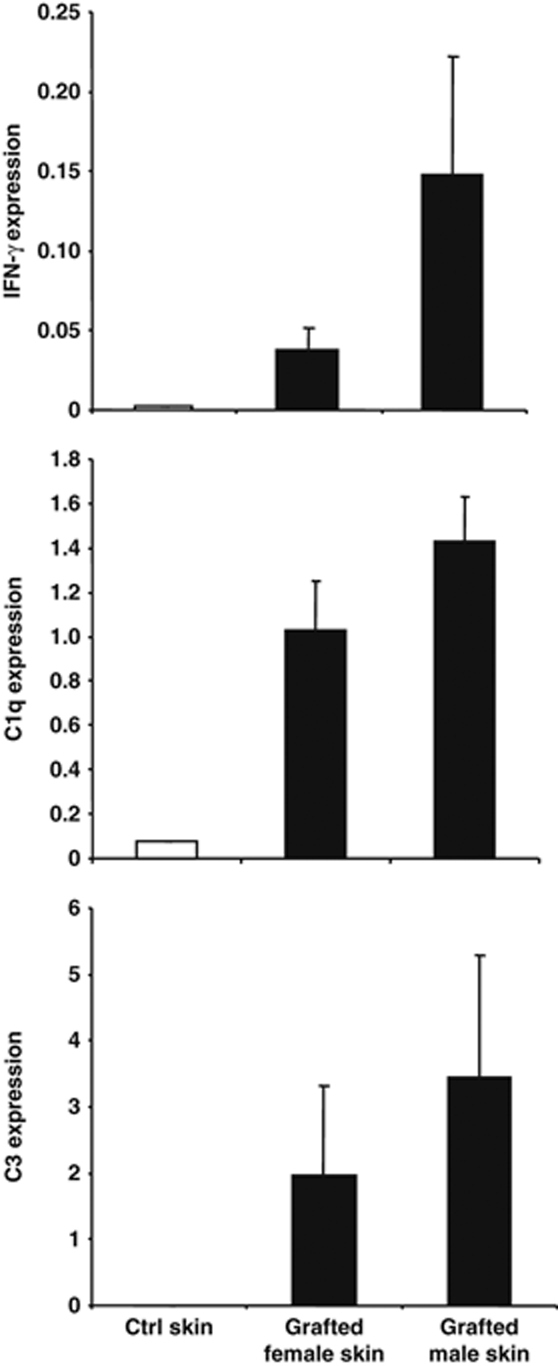
Up-regulation of mRNA expression of IFN-γ, C1q and C3 in tolerated male grafts. HY^Ab^*Dby* peptide was administered intranasally to WT female mice followed by grafting of WT male and female skin. The skin grafts were harvested 3 months later and analyzed using quantitative PCR analysis of IFN-γ, C1q and C3 mRNA. Ungrafted female tail skin was used as control. The *y*-axis represents an arbitrary linear scale of the mean ± SEM mRNA products in skin samples. The results represent pooled data from skin grafts of three mice.

We next tested whether local production of complement proteins and IFN-γ occurs following induction of tolerance. WT female mice were given intranasal peptide and grafted with syngeneic male and female skin. Both grafts were harvested from the tolerized mice 10−12 wk after grafting and the mRNA expression of C1q, C3 and IFN-γ analyzed in the grafted skin. Tail skin from WT female mice were used as controls. The expression of IFN-γ, C1q and C3 mRNA was found to be several fold higher in the skin grafts compared with control female skin (C1q mRNA: 1.03±0.22 female graft *versus* 1.43±0.2 male graft, expressed in arbitrary units; C3 mRNA: 1.97±1.35 female graft *versus* 3.45±1.84 male graft; IFN-γ mRNA: 0.04±0.01 female graft *versus* 0.15±0.57 male graft) (Fig. 5).

## Discussion

The lack of complement is strongly linked with development of autoimmune phenomena. This is particularly true for the early components of the classical complement pathway 25,26. Deficiency of C1q can lead to SLE-like phenomena in both humans and mice, suggesting that C1q is an important contributor to maintaining self-tolerance. However, the role of C1q in “tolerance” to foreign tissue remains largely unexplored. Here, we demonstrate that C1q indeed contributes toward tolerance induction and/or maintenance of grafted foreign tissue. First, female mice grafted with syngeneic male skins reject their grafts much faster in the absence of C1q. Second, intranasal administration of HY peptide fails to induce tolerance to male skin in the absence of C1q.

Interestingly, we also show that deficiency of C3 results in a rapid rejection of male skin grafts, similar to that observed with C1q deficiency. Tolerance to male grafts following intranasal administration of HY peptide also fails to occur when C3 is absent. This effect observed with C3 on HY mismatched transplants is perhaps surprising given the previous literature on the role of C3 in MHC-disparate allo-transplantation. Pratt *et al*. have demonstrated that MHC-disparate renal allografts survive longer in the absence of C3 9. Also, other studies have implicated complement in the rejection of both MHC-mismatched allografts and xenografts 9,27. In this context, it is notable that the major difference between our observations and the previous published data 9,27 relate to the different nature of the grafts. Kidney 9 and heart transplantation 27 involves grafting of a vascularized solid organ, a very different procedure to skin grafting. Furthermore, MHC-mismatched transplants induce antibody responses that could account for the differences observed between our study and previous publications. In support of the hypothesis that different experimental models underlie the differences, a requirement for C3 in the induction of tolerance has also been reported in some models. An up-regulation of C3 has been noted in the transplanted rat liver following induction of tolerance 28. Also, C3 has been shown to be involved in the suppression of a delayed type-hypersensitivity response to antigen injected into the anterior chamber of the eye 4.

The exact mechanism by which the failure of tolerance induction occurs in the C1q and C3-deficient mice is yet to be understood. Rejection of HY mismatched skin grafts is primarily a T-cell-driven effect 29. We have previously shown that peptide-induced tolerance causes reduction in the number of antigen-specific HY D^b^*Uty* specific CD8^+^ T cells, but does not eliminate them entirely 24. In keeping with this, an increase in HY peptide specific CD8^+^ T cells was noted both in the circulation and in the DLN of peptide pretreated C1qa^−/−^ and C3^−/−^ mice rejecting the male grafts and this appears to correlate with graft rejection. This suggests that C1q and C3 influence the graft rejection by modulating antigen-specific T cells. This is not surprising as both C1q and C3 have been demonstrated to impact on the function of antigen presenting cells and T cells *in vitro* 12–15. Furthermore, in our model during the induction phase of tolerance (prior to skin grafting), the administration of peptide does not lead to any detectable expansion of endogenous antigen-specific CD4^+^ T cells. This contrasts to administration of peptide plus LPS (which causes accelerated graft rejection) that expands the endogenous population of antigen-specific CD4^+^ T cells producing both IFN-γ and IL-2 24. Subsequent adoptive transfer experiments, using naïve antigen-specific CD4^+^T cells, have revealed that in peptide tolerized WT animals there is an initial expansion of antigen-specific CD4^+^ T cells that have upregulated FoxP3, LAG3, CTLA4 and IFN-γ (our unpublished data). As C1q and C3 have been shown to be required for the optimum production of IFN-γ by antigen-specific T cells 12,15, one could hypothesize that the inadequate local IFN-γ production in C1qa^−/−^ and C3^−/−^ mice may contribute to the failure of HY peptide to generate this population of regulatory T cells, an hypothesis consistent with the published *in vitro* findings. It would be also consistent with the finding that, following male skin graft, the T cells accumulating in the DLN of C1q- and C3-deficient mice that have rejected the male grafts produced more IFN-γ and, curiously, more IL-10, following *in vitro* restimulation with HY peptide pulsed APC. These cytokines may just reflect the ongoing inflammation or may directly contribute to mediate rejection in HY-disparate grafting in the context of complement deficiency, by supporting the expansion of CD8^+^ effector T cells. Our previous findings of increased numbers of antigen induced IL-10 as well as IFN-γ-producing T cells in non-tolerant controls in comparison with HY peptide induced skin graft tolerant mice 24 are also consistent with this. The presumption here would be that IL-10 could be produced by Th1 cells for self-regulation. More work will be needed to dissect the events through which a deficiency of C1q or C3 results in the accumulation of antigen-specific T cells *in vivo*. Recently, deficiency of C1q was shown to associate with a rapid rejection of MHC-mismatched cardiac allografts 30. In this model, rejection was found to associate with accelerated humoral responses to alloantigens in C1qa^−/−^ mice but not with enhanced T-cell responses.

Another possible mechanism for failure of intranasal tolerance induction in complement-deficient mice could involve antigen presentation *per se*. Induction of tolerance by intranasal administration of the MHC class II–restricted HY^Ab^*Dby* peptide is likely to be the result of peptide presentation by DC in the absence of a second immunostimulatory signal. We and others have shown that C1qa^−/−^ or C3^−/−^ DC are poorer than WT DC in presenting antigen to antigen-specific T cells 12–15. Thus, ineffective presentation of intranasal peptide in C1q- and C3-deficient mice could prevent the development of the antigen-specific CD4^+^ T cells expressing regulatory phenotype that are thought to be required for subsequent tolerance.

Local production of factors in the grafted tissue may also contribute to tolerance induction. We found a relative up-regulation of C1q and C3 in the male skin grafts in the tolerized recipients compared with the female grafts. This observation is similar to the finding that C3 is up-regulated in tolerated liver grafts in a rat model 28. Interestingly, local levels of IFN-γ were also found to be higher in the tolerated male skin graft compared to the adjacent female skin graft. This is consistent with the observation that IFN-γ may have a paradoxical protective role in context of transplantation 31. IFN-γ has been shown to contribute to the function of regulatory T cells 32, as well as be a protective factor in an experimental autoimmune glomerulonephritis model 33. In addition, IFN-γ signaling on DC can induce indoleamine 2,3-dioxygenase expression, and indoleamine 2,3-dioxygenase-expressing DC can either promote the expansion of naturally occurring regulatory T cells or convert naïve CD4^+^ T cells into induced regulatory T cells 34. We have recently shown that C1q is required for the optimum production of IFN-γ by antigen-specific T cells 12,15. Similarly, C3 deficiency associates with a decreased production of IFN-γ by T cells. Thus inadequate local IFN-γ production in C1qa^−/−^ and C3^−/−^ mice may well contribute to rapid rejection of the male grafts and to the failure of tolerance induction with HY peptide.

These novel results highlight a new facet to complement immunology. In addition to the tolerization to HY disparate grafts, intranasal administration of peptide has been used to induce tolerance in murine models of autoimmunity as well as asthma 35,36. Thus the requirement of C1q and C3 for promoting intranasal tolerance is a new finding with therapeutic potential. Future research directed toward dissecting the mechanisms by which complement influences the balance between graft tolerance/rejection could firmly place complement in the armamentarium of the transplant immunologist.

## Materials and methods

### Culture media and reagents

All cells were cultured in RPMI 1640 (Invitrogen Life Technologies) containing 100 U/mL penicillin, 100 μg/mL streptomycin, 1.5 mM l-glutamine and 10% heat-inactivated FBS.

### Mice

BALB/c and B6 mice (8–12 wk old) were purchased from Harlan (Blackthorn, UK). B6.C1qa^−/−^ 18, B6.C3^−/−^, BALB/c.C1qa^−/−^ and BALB/c.C3^−/−^ were generated by backcrossing the original strain onto the C57BL/6 or BALB/c genetic background for ten generations.

### Peptides and tetramers

Peptides HY^Ab^*Dby*, NAGFNSNRANSSRSS; HY^Db^*Uty*,WMHHNMDLI were prepared as previously described 24. MHC class I tetramers were produced using a previously described 37 modification of the method of Altman *et al*. 38.

### Quantitative RT-PCR

RNA was prepared from tail skin using mechanical homogenization and TRIzol reagent (Invitrogen Life Technologies) as previously described 39. Test samples were assessed in triplicate for expression of 18S ribosomal RNA and IFN-γ, C1q and C3 transcripts. Expression values were determined from standard curves generated from each RNA preparation, plotting cycle threshold values against log quantity. Normalized IFN-γ, C1q and C3 values were obtained by division with the corresponding 18S value and expressed in arbitrary units.

### PBL staining

Mice were tail-bled into tubes containing EDTA. Red blood cell lysis was performed and samples centrifuged. The cells were washed with PBS and pelleted. The pellet was resuspended in PBS 1% FBS and incubated for 10 min at 37°C with CD8-FITC and HY^Db^*Uty* tetramer–PE. Cells were washed and measured using flow cytometry.

### *In vitro* expansion of tetramer-positive T cells isolated from DLN

Axillary nodes from the side of grafted skin were collected and single-cell suspensions were prepared. The DLN cells (4×10^6^/well) were cultured with irradiated syngeneic male spleen cells (4×10^6^/well) and 10 IU/mL recombinant human IL-2 (hu-rIL-2) in 24-well plates. Cultures were sampled on day 7 for tetramer-specific T-cell analysis as above. The remaining cells were then used for quantification of intracellular cytokines as detailed below.

### Intracellular cytokine detection/quantification

To stimulate cytokine production, splenocytes (4×10^6^/mL) or DLN cells from graft-rejected or tolerant WT, C1qa^−/−^ and C3^−/−^ B6 females were cultured with irradiated B6 male splenocytes (4×10^6^/mL), in the presence of hu-rIL-2 (10 IU/mL). Seven days later, the activated T cells (2×10^6^/mL) were re-stimulated with irradiated unpulsed or peptide-pulsed B6 female T-cell-depleted splenocytes (4×10^6^/mL) for 6 h in the presence of monensin (GolgiStop; BD Biosciences Pharmingen). Cultured cells were incubated with 2.4G2 antibody, washed and stained with anti-CD4–PerCP and anti-CD8–PE. After washing, cells were fixed, permeabilized with Cytofix/Cytoperm solution (BD Biosciences Pharmingen), washed with Perm/Wash solution (BD Biosciences Pharmingen) and resuspended in Perm/Wash solution containing anti-IFN-γ–FITC and anti-IL-10–APC or isotype-matched control antibodies. The cells were then washed and resuspended in staining buffer for flow cytometry analysis.

### Intranasal peptide administration

PBS (20 μL) containing 100 μg HY^Ab^*Dby* peptide was administered intranasally on three consecutive days to WT, C1qa^−/−^ and C3^−/−^ B6 female mice following anesthesia with isoflurane. Control mice did not receive the intranasal peptide. The mice were grafted with syngeneic male and female skin grafts 10 days later.

### Skin grafting

Skin grafting was conducted by the method of Billingham and Medawar using tail skin grafted onto the lateral thorax. After removal of the plaster casts, grafts were observed every 2 to 3 days and scored as rejected when less than 10% viable tissue remained 24.

### ELISA

A sandwich ELISA for IFN-γ was performed using purified capture and biotinylated detection antibodies from BD-Pharmingen. The samples and standards were added in duplicates.

### Statistical analysis

Statistical analysis was performed using the two-tailed Student's *t*-test for unpaired samples with unequal variance. Probability values (*p*) less than 0.05 were considered statistically significant. Statistical analysis for survival curves was performed using the log-rank method in GraphPad Prism version 3.02 software (San Diego, CA, USA).

## Abbreviations

DLN: draining LN
MST: mean survival time
