# Supplementary material for: Mice lacking C1q or C3 show accelerated rejection of minor H disparate skin grafts and resistance to induction of tolerance
Source: Eur J Immunol. 2010 Mar 8;40(6):1758–67. doi: 10.1002/eji.200940158 (PMC2988415; doi:10.1002/eji.200940158)
Supplement: Supplementary file 1 [file eji0040-1758-SD1.pdf]

European Journal of Immunology

Supporting Information

for

DOI 10.1002/eji.200940158

Mice lacking C1q or C3 show accelerated rejection of minor H disparate skin grafts and resistance to induction of tolerance

Paramita Baruah, Elizabeth Simpson, Ingrid E. Dumitriu, Katy Derbyshire, David Coe, Caroline Addey, Julian Dyson, Jian-Guo Chai, Terence Cook, Diane Scott and Marina Botto

© 2010 WILEY-VCH Verlag GmbH & Co. KGaA, Weinheim

Baruah et al - Supplementary Figure 1

Non peptide treated group

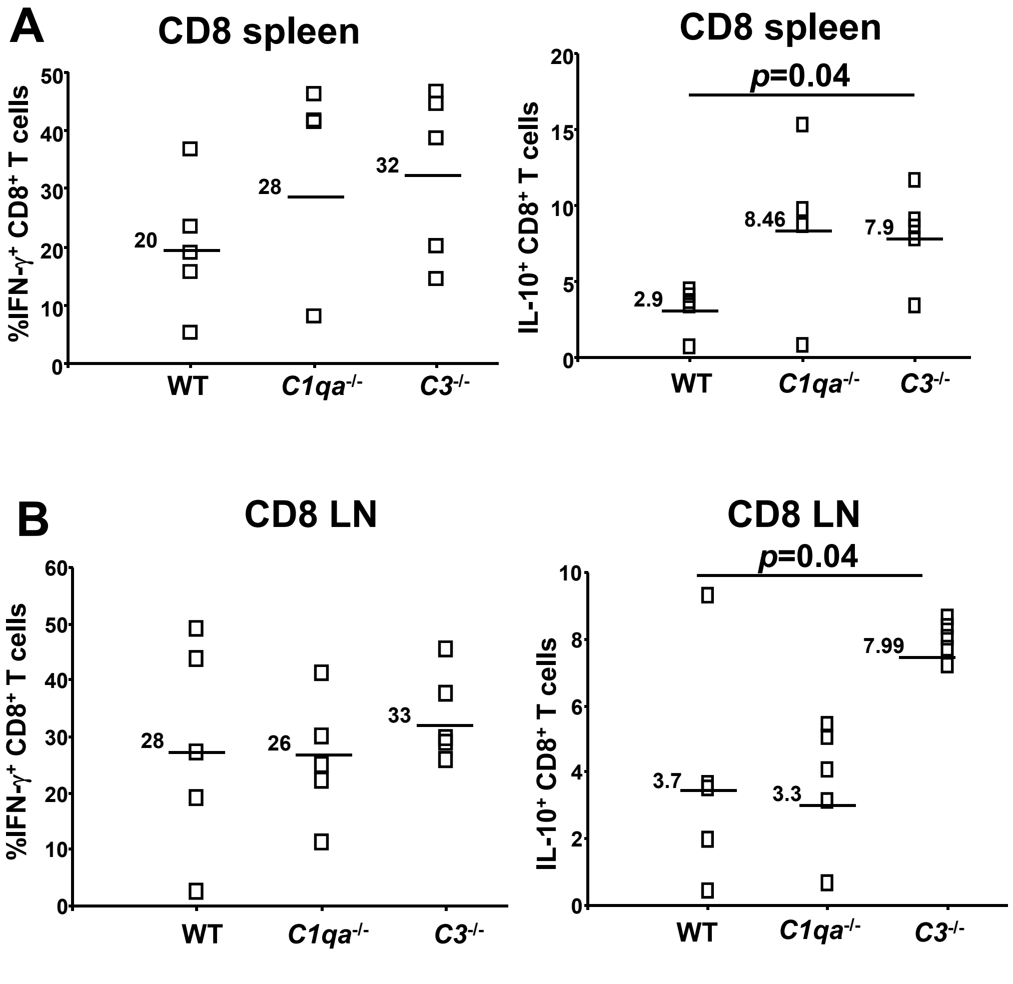

Supplementary Figure 1. *Ex vivo* cytokine responses following skin grafting. WT $C1qa^{-/-}$ and $C3^{-/-}$ male skin was grafted on WT, $C1qa^{-/-}$ and $C3^{-/-}$ mice. Following an intraperitoneal boost with male splenocytes 12 weeks later, the mice were sacrificed and cells from DLN and spleen harvested. Cells isolated from the DLN and spleen were cultured in vitro with irradiated male splenocytes and then restimulated with HY^{Db}*Uty*-peptide-pulsed female splenocytes. A. IFN-γ producing $CD8^+$ T cells and IL-10 producing $CD8^+$ T cells from spleen were quantified by intracellular staining and flow cytometry. B. IFN-γ producing $CD8^+$ T cells and IL-10 producing $CD8^+$ T cells from DLN were quantified by intracellular staining and flow cytometry. $p<0.05$, statistically different from control.
